# Turkish preschoolers show an advantage in their understanding of their own representational change over others' false belief

**DOI:** 10.1111/bjdp.70050

**Published:** 2026-05-13

**Authors:** Mesut Saçkes, Sonnur Işıtan, Kathy Cabe Trundle, David M. Sobel

**Affiliations:** ^1^ Balikesir University Balikesir Turkey; ^2^ Utah State University Logan Utah USA; ^3^ Brown University Providence Rhode Island USA

**Keywords:** false belief, representational change, theory of mind, Türkiye

## Abstract

Recent studies suggest that Turkish preschoolers exhibit a different pattern of theory of mind development than Western samples, particularly with respect to understanding the diversity of beliefs and knowledge acquisition. The present study posits that such differences extend to distinctions between understanding others' false belief and one's own representational change. Across two experiments, we found that preschoolers in Türkiye (*n* = 493; 249 Girls and 244 Boys) showed superior performance when asked a question about their own representational change compared with their understanding of another's false belief. These findings support the hypothesis that Turkish children attend to different facets of their own mental states, which might influence reasoning about their own representational change differently from others' false belief.

**Statement of contribution**

**What is already known on this subject?**

- Meta‐analyses of predominantly Western, English‐speaking samples suggest that children's understanding of their own representational change and others' false beliefs typically develops concurrently, with few significant performance differences between the two.
- Turkish‐speaking children may have a linguistic advantage in Theory of Mind tasks due to obligatory evidential markers and specific mental state verbs, such as sanmak (falsely think), which explicitly denote mistaken beliefs.
- Turkish children master the concept of knowledge access before understanding diverse or conflicting beliefs, potentially due to cultural emphases on factual accuracy.

**What the present study adds?**

- Turkish preschoolers show a significant advantage in understanding their own representational change compared to others’ false beliefs.
- This “self‐advantage” remains consistent regardless of the specific mental state verb used (düşünmek vs. sanmak) or the order and temporal framing of the questions.
- Turkish linguistic and cultural environment, which prioritizes source‐monitoring and epistemic certainty, may scaffold the tracking of one's own shifting knowledge states more effectively than the attribution of subjective false beliefs to others.

The development of a representational theory of mind supports the understanding that others' beliefs can conflict with reality (e.g. Perner, 1991; Wellman, 1990; Wimmer & Penner, 1983), as well as the understanding that one's own representations of beliefs can change with new information (e.g. Gopnik & Astington, 1988; Perner et al., 1987). As an example, in the *unexpected contents* task, children are shown a familiar container (such as a crayon box) and asked what is inside. After revealing that children understand the container's identity (i.e. they say that crayons are in the box), the box is revealed to be deceptive (e.g. it really contains birthday candles). The deceptive contents are returned to the box, which is then sealed. Children are then asked a question about another's false beliefs (the *other* question, e.g. ‘What will someone else think is in the box?’) as well as a question about their own representational change (the *self*‐question, e.g. ‘Before I showed you what was inside, what did you think was in the box?’). In general, performance on these questions improves between the ages of 3 and 5 years (Wellman et al., 2001).

Although early studies of false belief and representational change found differences in preschoolers' performance on these two questions (e.g. Gopnik & Astington, 1988; Perner et al., 1987), using meta‐analytic techniques, Wellman et al. (2001) found that performance on these questions did not differ significantly. In an updated meta‐analysis, Sobel (2023) found an interaction between question type (self‐ vs. other or own representational change vs. others' false belief) and age. Three‐year‐olds performed better on questions about their own representational change than on questions about others' false beliefs; 4‐year‐olds showed the opposite pattern. These analyses suggest that children's developing understanding of their own representational change and others' false belief might have distinct developmental trajectories. This conclusion, however, was based on reviews of studies with samples of children who spoke predominantly Indo‐European languages and were members of Western, Educated, Industrialized, Rich and Democratic (WEIRD) societies, as well as a mega‐analysis of data from exclusively English‐speaking children in the United States.

The present study extends this line of research to a Turkish sample. Studying children in Türkiye presents an opportunity to engage with the broader literature on theory of mind development in this community. Several researchers have proposed that Turkish‐speaking children may demonstrate superior performance on false‐belief tasks compared with children speaking other languages because of two unique linguistic features of Turkish (referred to as the *Turkish Advantage* hypothesis, e.g. Aksu‐Koç & Alici, 2000; Aksu‐Koç et al., 2009; Granti, 2004). First, Turkish grammar requires the presence of obligatory evidential markers, which require speakers to indicate the source of their knowledge (e.g. firsthand experience vs. hearsay), potentially fostering an early awareness of the difference between beliefs and reality (Aksu‐Koç, 1988; Lucas et al., 2013; Shatz et al., 2003). Second, Turkish has distinct mental‐state verbs, such as *sanmak* and *zannetmek* that directly denote holding a mistaken belief. These verbs can contrast with a verb like *düşünmek*, which more generally refers to thinking without indicating an epistemic value to the content (Aksu‐Koç et al., 2009; Haskaraca & Ilgaz, 2023; Lucas et al., 2013; Shatz et al., 2003).

Preschoolers in Türkiye do show higher rates of success on the unexpected contents task (combining performance on the self‐ and other questions) when the mental verb ‘sanmak’ (falsely think) was used over the more epistemically neutral verb ‘düşünmek’ (e.g. Aksu‐Koc et al., 2005; Granti, 2004). Turkish children also outperform peers from other language backgrounds on other measures of false belief, such as the unexpected transfer task (e.g. Kandemirci et al., 2023; Lucas et al., 2013; Shatz et al., 2003). Turkish children's performance on questions about others' false beliefs compared with questions about other mental states, such as knowledge access, even when the verb for falsely think is used (Haskaraca & Ilgaz, 2023) or when obligatory evidential markers are present (Özoran, 2009; see also Çelik et al., 2024; Erden Ozcan & Ozgun, 2024; Etel & Yagmurlu, 2015).

More generally, Selcuk et al. (2018) suggested that Turkish children exhibited a ‘collectivist acquisition pattern’ (p. e2098) in theory of mind development using the theory of mind scales (Wellman & Liu, 2004), similar to children in China (Wellman et al., 2006) and Iran (e.g. Shahaeian et al., 2011). Children in Western cultures understand that others can have different beliefs about the same situation before they understood the relation between perceiving the contents of a box and knowing what's inside. By contrast, children in these cultures show the reverse developmental pattern (i.e. an understanding of knowledge access prior to an understanding of diverse beliefs). This difference is development reflects the potential importance placed in understanding one's own and others' knowledge (as reflected in, among other things, the complex evidential system that children are developing in Turkish).

Few studies have examined Turkish children's understanding of changes in their own knowledge in isolation (i.e. how children perform on measures of representational change independent from measures of false belief), but those that do suggest strong performance (e.g. Aksu‐Koc et al., 2005; Granti, 2004; Kaysılı & Acarlar, 2011). Kaysılı and Acarlar (2011) in particular found that Turkish 3‐year‐olds showed a better understanding of representational change than others' false belief. Furthermore, they showed that performance on measures of others' false belief improved with age, while performance on a representational change question did not. These studies provide initial evidence that Turkish children might perform better on measures of representational change than measures of false belief. However, these studies had small samples—only 24 children per age group—which suggests they might not have been sufficiently powered. Moreover, these studies only tested children with the verb falsely think (i.e. sanmak), and did not contrast children's performance between using such a verb, where one might expect improvement, and a verb like think (düşünmek), where the truth‐value of the belief conveyed is uncertain.

The two studies presented here consider a larger‐scale investigation of Turkish children's performance on false belief and representational change questions in an unexpected contents task. The first study establishes a performance difference between the two questions. The second study then reproduces this finding using a more tightly controlled procedure.

## STUDY 1

### Method

#### Participants

A total of 316 children (158 girls and 158 boys, *M* age = 53.67 months, SD = 5.13 months, range 40–61 months) participated in the study. Children were recruited from 10 preschools serving predominantly middle‐income families in a midsize city in north‐western Türkiye. All children were native Turkish speakers. Informed consent forms were collected from the parents. Verbal assents from the children were obtained prior to data collection. The study was approved by the Balikesir University Social Sciences and Humanities Ethics Commission (Approval No 2021/02).

#### Materials and procedure

The researchers administered a modified version of an unexpected contents task (Gopnik & Astington, 1988; Perner et al., 1987) to the child individually in a quiet room at the child's preschool. The researcher introduced a locally well‐known chocolate box to the child. The child was asked to guess the contents of the box. All children responded with chocolate or an appropriate synonym. The researcher then revealed that there were crayons inside the box instead of chocolates. The child was allowed to touch the box and its contents. The crayons were then placed back in the box, which was then closed. Children were then asked questions about whether another child would have a false belief about the content of the box and about the child's own representational change.

Because Turkish has verbs for both falsely think (*sanmak*) and think (*düşünmek*), different subsets of the sample were asked about their own representational change (i.e. the self‐question) and another's false belief (the other question) with one of three wordings (shown in Table 1). One subset of the sample (*N* = 72) was asked both the self‐ and other questions using the verb düşünmek. A second subset of the sample was asked both the self‐ and other questions using the verb sanmak (*N* = 80). For these two subsets, when children were asked the other question, they were shown a picture of a boy (Bilge) or a girl (Gökçe), gender matched to the child. They were told that the character did not see when the researcher and child opened the chocolate box, did not hear what they talked about, and had never seen the box or its contents before. The third subset of the sample (*N* = 164) was asked the self‐question using the verb sanmak in the same manner as above. For the other question, children were introduced to a novel plush toy that was sleeping while the researcher and child interacted with the chocolate box, and did not see, hear or know about its contents. Children were asked, ‘If I ask the toy what is inside this box, how would s/he respond?’ (*cevap verirdi*). This wording was used to focus solely on the character's behaviour, rather than asking children about mental states. ‘This specific verb is more neutral than *falsely think* and less abstract for Turkish children than *think*’ (A. Aksu‐Koç, personal communication, 19 February 2014).

**TABLE 1:** Exact wording of the test questions for the three subsets of children.

Subsets	Self‐question	Other question
Subset 1: ‘Think’ for both self‐ and other questions	Kutuyu ilk gördüğünde, açmadan önce, kutunun içinde ne olduğunu düşündün? *When you first saw the box, before you opened it, what did you think was inside the box?*	Bilge/Gökçe kutuyu gördüğünde içinde ne olduğunu düşünür? *When Bilge/Gökçe sees the box, what does he/she think is inside?*
Subset 2: ‘Falsely think’ for both self‐ and other questions	Kutuyu ilk gördüğünde, açmadan önce, kutunun içinde ne olduğunu sandın? *When you first saw the box, before you opened it, what did you falsely think was inside the box?*	Bilge/Gökçe kutuyu gördüğünde içinde ne olduğunu sanır? *When Bilge/Gökçe sees the box, what does he/she falsely think is inside?*
Subset 3: ‘Falsely think’ for self‐question, ‘would respond’ for other question	Kutuyu ilk gördüğünde, açmadan önce, kutunun içinde ne olduğunu sanmıştın? *When you first saw the box, before you opened it, what did you falsely think was inside the box?*	Şimdi oyuncağa bu. kutunun içinde ne olduğunu sorsam, bana ne cevap verirdi? *Now, if I ask the toy what is inside this box, what would s/he respond to me*

Based on our conversations with teachers in our testing settings, this wording is the most pragmatically appropriate way to ask about another's false belief, particularly given that it resembles adult‐child conversational routines used in Turkish preschool contexts. In contrast, for the self‐question, ‘sanmak’ was more epistemically and pragmatically appropriate; the box had been opened, and it was evident that children's initial statement about the content of the box was not true. The use of this verb signalled to children that their guess did not match the actual contents of the box. To our knowledge, however, this is the only study that directly contrasts the verbs used to ask these questions (and particularly the self‐question) in Turkish, a point we will return to in the discussion. Finally, children were asked a control question, namely, whether the subject of the other question had seen the contents of the box.

### Results and discussion

Children were scored as responding correctly if they stated that they initially believed that candy was in the box (self‐question) or that the toy/kid would respond that candy was in the box (other question), as long as they responded correctly on the control question.^1^ Otherwise, they were scored as not responding correctly. Table 2 shows children's performance on the self‐ and other questions based on the wording used to ask the questions for both this study and Study 2.

**TABLE 2:** Percentage of children responding correctly on the representational change (self) and false belief (other) questions based on the wording used in Study 1 and 2. Standard deviation in parentheses.

	Think (both questions)	Falsely think (both questions)	Falsely think (self), would respond (other)	Total
Self‐question Study 1	43% (50)	68% (47)	83% (38)	70% (46)
Other question Study 1	29% (46)	49% (50)	24% (43)	31% (46)
Self‐question Study 2 (all data)	66% (47)	74% (44)	N.A.	69% (46)
Other question Study 2 (all data)	51% (50)	55% (50)	N.A.	53% (50)
Self‐question Study 2 (4‐year‐olds only)	47% (50)	67% (47)	N.A.	56% (50)
Other question Study 2 (4‐year‐olds only)	30% (46)	50% (50)	N.A.	40% (49)

We compared performance on the self‐ and other questions via a generalized linear mixed model assuming a binomial logistic distribution. Age (in months), question type (self vs. other), and the three wordings were the fixed effects, and participant ID was the random effect. A model with all main effects and interactions was considered. This analysis revealed a significant effect of age, *β* = 0.13, SE = 0.03, *t* = 3.83, *p* < .001, and a significant interaction between question type and question wording, *χ* ^2^(2) = 37.57, *p* < .001. This interaction resulted from the difference in performance between the self‐ and other questions in the third subset of the sample (self‐question asked with falsely think, other question asked with would respond, 83% vs. 24%) being greater than the second subset (both self‐ and other questions asked with falsely think, 68% vs. 49%), *β* = 0.38, SE = 0.18, *t* = 2.18, *p* = .03, and the first subset (both self‐ and other questions asked with think, 43% vs. 29%), *β* = 0.67, SE = 0.19, *t* = 3.54, *p* < .001. Controlling for age, performance on the other question in the third subset (would respond) was significantly lower than performance on the self‐question (falsely think), *β* = −2.06, SE = 0.52, *t* = −3.97, *p* < .001. This was also the case in the second subset (falsely think for both questions), *β* = −0.68, SE = 0.23, *t* = −2.91, *p* = .004, while the difference for the first subset (think for both questions) was not significantly different, *β* = −0.46, SE = 0.24, *t* = −1.90, *p* = .06. Controlling for age, performance on self‐question was better when the verb falsely think was used (77%) over the verb think (40%), *β* = 1.78, SE = 0.29, Wald *χ* ^2^(1) = 35.44, *p* < .001. In contrast, the three methods of asking the other question were not significantly different, Wald *χ* ^2^(2) = 5.60, *p* = .06.

We next compared performance to that of chance responding (50%). Overall, performance on the self‐questions (70%) was significantly higher than what would be expected by chance, binomial test, *p* < .001, and performance on the other questions (31%) was significantly lower than what would be expected by chance, binomial test, *p* < .001. When we considered which wording was used in the other question, overall performance with the wordings ‘think’ and ‘would respond’ was below chance, with both binomial tests yielding *p*‐values <.001. Performance with the wording ‘falsely think’ resulted in chance‐level performance (44%), *p* = .31. Even when only the 4‐year‐olds were considered, performance was still at chance levels (49%), binomial test, *p* = .91. In contrast, performance on the self‐question was greater than chance performance when the verb ‘falsely think’ was used (77%), binomial test, *p* < .001, but not significantly different from chance when ‘think’ was used (40%), *p* = .13.

The Turkish preschoolers investigated in this study showed relatively strong performance on the self‐question regarding understanding their own representational change and relatively weak performance on the other question regarding their understanding of others' false beliefs. The type of verb used to ask questions about others' false beliefs or the child's own representational change influenced performance, with the verb for falsely think resulting in better performance overall than the verb for think. However, across the ways the questions were asked, performance on the self‐question was better than chance and better than performance on the other questions, which were mostly below chance.

One concern with Study 1 was that, across all language conditions, children were asked the self‐ and other questions in a fixed order, with the self‐question always preceding the other question. It is possible that being asked the self‐question first affected responses to the other question negatively, causing the difference in performance. In addition, the self‐question was phrased with a temporal marker that prompted children to reason about the past; the other question in Study 1 lacked that temporal marker. Lewis and Osborne (1990) found that the presence of such temporal markers (i.e. ‘before I showed you what was inside the box…’) improved performance on representational change questions in a British sample of preschoolers. Thus, it is possible that children's superior performance on the self‐question in Study 1 resulted from the presence of this temporal language. Study 2 thus reproduced the investigation of differences between the self‐ and other question, asking the question either with the verb falsely think (*sanmak*) or think (*düşünmek*), counterbalancing the order in which the two questions were asked and ensuring that both questions had this temporal marker.

## STUDY 2

### Method

#### Participants

The final sample consisted of 177 children (91 girls and 86 boys; *M* age = 59.95 months, SD = 6.72 months, Range 48–71 months). Children were recruited from four preschools serving predominantly middle‐income families in a midsize city in north‐western Türkiye. All children were native Turkish speakers. Informed consent forms were collected from the parents prior to data collection. The study was approved by the Balikesir University Social Sciences and Humanities Ethics Commission (approval no.: 2025/01‐35).

#### Materials and procedure

The same materials as Study 1 were used here. The procedure for the unexpected contents task was similar to that of Study 1. Approximately half of the sample (*n* = 90) were tested with the verb ‘think’; the remaining children (*n* = 87) were tested with the verb ‘falsely think’. Within each group, approximately half of the children received the self‐question first (48/90 in the think condition; 42/87 in the falsely think condition); the other children received the other question first. The wording of the self‐question was similar to that of Study 1. The wording of the other question included the same temporal marker as the self‐question, to ensure that the language was as similar as possible. Table 3 shows the exact wording of the questions.

**TABLE 3:** Exact wording of the test questions for the children in Study 2.

Groups	Self‐question	Other question
‘Think’ for both self‐ and other questions	Kutuyu ilk gördüğünde, açmadan önce, kutunun içinde ne olduğunu düşündün? *When you first saw the box, before you opened it, what did you think was inside the box?*	Bilge / Gökçe kutuyu ilk gördüğünde açmadan önce kutunun içinde ne olduğunu düşünür? *When Bilge/Gökçe sees the box for the first time, before opening it, what he/she thinks is inside?*
‘Falsely think’ for both self‐ and other questions	Kutuyu ilk gördüğünde, açmadan önce, kutunun içinde ne olduğunu sandın? *When you first saw the box, before you opened it, what did you falsely think was inside the box?*	Bilge / Gökçe kutuyu ilk gördüğünde açmadan önce kutunun içinde ne olduğunu sanır? *When Bilge/Gökçe sees the box for the first time, before opening it, what he/she falsely thinks is inside?*

### Results and discussion

The representational change (self‐) and false belief (other‐) questions were scored in the same manner as in Study 1; children had to respond correctly to the test question and pass the control measure associated with that question. Table 2 presents the percentage of children who responded correctly to the self‐ and other questions across the two language conditions (see also Figure 1). We first considered the order in which the questions were asked. Overall, children responded appropriately to the self‐question when it was asked first 72% of the time, compared with 67% of the time when the other question was asked first; children responded appropriately to the other question 56% of the time when the self‐question was asked first and 49% of the time when the other question was asked first. Neither of these differences was significant, self‐question, *χ* ^2^(1, *N* = 177) = 0.64, *p* = .42, other question, *χ* ^2^(1, *N* = 177) = 0.93, *p* = .33.

**FIGURE 1:**
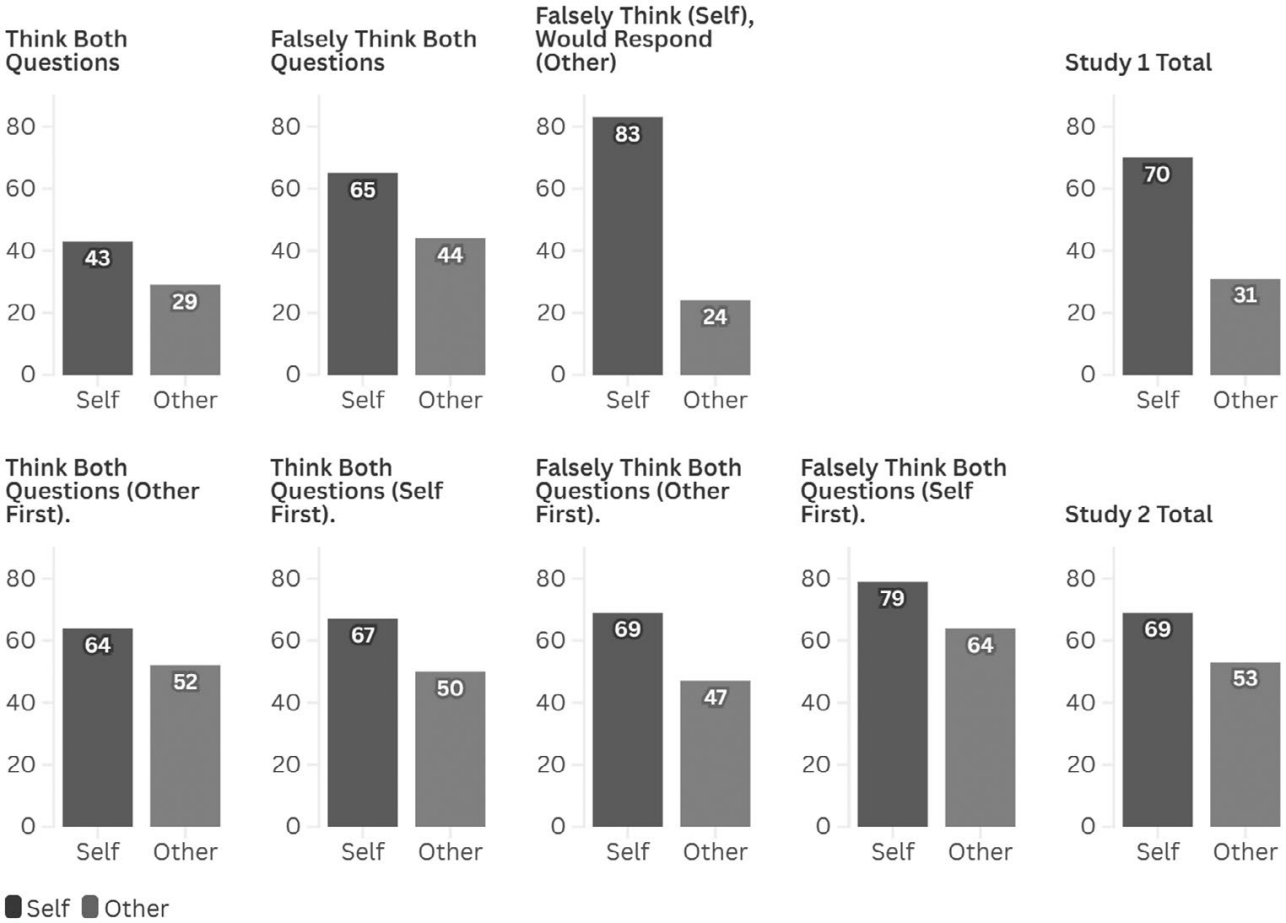
Children's performance on the self‐ and other questions in Studies 1 and 2.

Whether children responded appropriately on each question was analysed with a generalized linear mixed model, assuming a binomial logistic distribution. Age (centred, in months), question type (self vs. other) and the two wordings (think vs. falsely think) were fixed effects, and participant ID was a random effect. A model with all main effects and interactions was considered. This analysis revealed a main effect of age, *β* = 0.12, SE = 0.03, *t* = 3.91, *p <* .001, and a main effect of question type, with performance on the self‐question superior to that of performance on the other question, *β* = 0.54, SE = 0.15, *t* = 3.53, *p <* .001. No other significant main effects or interactions were found. Across the two wordings, children responded correctly on the self‐question 69% of the time, significantly above chance, binomial test, *p* < .001. In contrast, children responded correctly on the other question 53% of the time, not different from chance, binomial test, *p* = .45.

A difference between Study 1 and Study 2 is that Study 2 did not find any effect related to the wording of the question. Moreover, overall performance in Study 2 was greater than that of Study 1. However, a difference between the two studies was that the sample in Study 2 was older than that in Study 1, largely because of the time of year in which children were tested and the composition of the preschools. This difference in sample age may explain the distinction findings between the studies. When 5‐year‐olds were not included in the analysis, there was a main effect of wording; children who were asked the questions with the word ‘think’ were less accurate (39% correct overall) than children who were asked the questions with the word ‘falsely think’ (58% correct), *β* = −0.54, SE = 0.23, *t* = −2.37, *p =* .02. Importantly, in this subset, children still showed a difference in their responses to the self‐ (56% correct) and other (40% correct) questions, *β* = 0.52, SE = 0.20, *t* = 2.66, *p =* .008, and responses to the other question were below chance levels at a marginal trend level, binomial test, *p* = .053.

Study 2 thus reproduces the main finding of Study 1, which is that Turkish children showed a difference between performance on the representational change and false belief questions on an unexpected contents task, and this difference held when children were asked the question with the word ‘think’ as well as the word ‘falsely think’, when both questions had temporal markers that asked about when the child or the character first saw the container, and when the order of the two questions was counterbalanced.

## GENERAL DISCUSSION

Across two studies, Turkish children's performance on measures of representational change was superior to analogous performance on measures of false belief. This finding held regardless of what mental state verb was used to ask the questions. These studies add to a growing body of literature suggesting that questions about others' false beliefs are challenging for Turkish children (e.g. Doenyas et al., 2018; Erden Ozcan & Ozgun, 2024; Ertuğrul‐Yaşar, 2024; Etel & Yagmurlu, 2015; Kahraman, 2012; Saçkes et al., 2026). Moreover, they also suggest that preschoolers in Türkiye are more capable of accurately answering questions about their own changes in beliefs. We speculate that Turkish children might have a better understanding of their own knowledge (including when it changes), but that understanding of their own knowledge does not generalize to cases where others have a false belief.

What is missing from this discussion is the mechanism for why children in Türkiye show differences in their performance between the self‐ and the other questions. Theory of mind development should be assessed within a cultural framework (e.g. Lillard, 1998). As such, we want to suggest three hypotheses, which may all be working in conjunction. First, Turkish children are socialized to prioritize factual accuracy and evidential grounding; they process questions about their prior thoughts through the lens of their information source history (Haskaraca & Ilgaz, 2023; Selcuk et al., 2018, 2023). This developmental pattern is reinforced by cultural socialization practices that prioritize factual accuracy over subjective internal states (e.g. Kagitcibasi & Ataca, 2005; Selcuk et al., 2018, 2023). Turkish parents often emphasize ‘accurate knowing’ and evidence‐grounded information, often viewing unfounded beliefs as mere ‘guesses’ (Haskaraca & Ilgaz, 2023; Kagitcibasi & Ataca, 2005). Such socialization encourages Turkish children to view the mind as a repository of information sources that must be accurately tracked rather than a generator of subjective representations. This cultural emphasis on epistemic certainty may explain why Turkish children excel at monitoring their own access to information while showing a delay in understanding the diverse beliefs measure as well as false beliefs in others, as these require shifting from witnessed knowledge to the more abstract realm of conflicting subjective perspectives (Selcuk et al., 2018, 2023; Wellman et al., 2006).

The observed performance gap between the self‐ and other questions demonstrated in these studies underscores the possibility of a knowledge‐first trajectory in the development of theory of mind in Turkish children (e.g. Phillips et al., 2021). Although Turkish children rely on well‐practised source‐monitoring skills to recall their own prior knowledge states, they may struggle with other‐oriented belief tasks that require navigating conflicting, subjective perspectives without direct linguistic or cultural scaffolding (e.g. Wellman et al., 2006). The specific linguistic and cognitive mechanisms of the Turkish language might prioritize a ‘knowledge‐first’ pathway to representational reasoning. Recognizing culture‐ and language‐specific epistemic features is essential for examining the diversity of cognitive development trajectories across different linguistic and cultural environments (Phillips et al., 2021; Selcuk et al., 2023).

Second (and related) Turkish children are developing a complex, obligatory evidential system that emphasizes tracking their own knowledge. We did not manipulate the evidential markers in our questions to children, primarily because children learning Turkish do not master the use of all such markers until approximately age 6 (Aksu‐Koç et al., 2009; Aksu‐Koç & Slobin, 1985). However, they do begin to produce the evidential marker of ‐DI (an indicator that knowledge is based on direct perception) around the age of 18 months and ‐MIŞ (an indicator that knowledge is based on inference) around the age of 24–30 months. By the age of 3, they appropriately use these markers in their communication (Ögel‐Balaban et al., 2012).

The early acquisition of the evidential marker ‐DI (indicating direct perception of experienced or witnessed events) might provide a linguistic format that is used in the encoding of experiences in memory (Aksu‐Koç et al., 2009). Combined with Turkish children's precocious competence of Knowledge Access (e.g. Selcuk et al., 2018), the development of this evidential system suggests that shared linguistic and cognitive mechanisms facilitate children's early mastery of self‐knowledge states (Granti, 2004; Kaysılı & Acarlar, 2011; Saçkes et al., 2026). This linguistic obligation provides children with a mental tag for their epistemic states, habituating them to monitor how they acquired specific information. In the context of an unexpected content task, this scaffolding allows children to track their initial state of accuracy, ignorance or inference more effectively. Consequently, when reality is revealed (the box contains an item other than candy), Turkish children can update their representations by recalling their history of knowledge access rather than reasoning about more abstract belief states. This representational updating capacity transforms the representational change question from a measure of abstract representational reasoning into one of source monitoring and memory retrieval. Consistent with the interpretation by Perner et al. (1987), Turkish children potentially process the self‐question (e.g. ‘What did you think was inside?’) as a query regarding the factual source of their previous mental state. Because the language necessitates constant attention to information sources, Turkish children become highly efficient at tracking their own witnessing of events (Kandemirci et al., 2023). This might allow them to successfully respond to the self‐question before their ability to attribute false beliefs to others, as the latter demands a more abstract understanding of subjective mental states (see also Lucas et al., 2013; Perner, 1991). Future research should consider whether there is a relation between comprehension and production of these particular evidential markers and performance on the unexpected contents task. If the development of these markers is a factor in children's performance, then one would predict a correlation with performance on the self‐question, but not the other question.

A third—and to our knowledge—unexplored hypothesis is that Turkish children might interpret questions about others' false belief as not satisfying the condition of plausible dissent (Crain et al., 1996), particularly when the more neutral verb for ‘think’ is used. The experimenter and child both know that Bilge/Gökçe does not know the actual content of the box and thus has a false belief. Children might ask themselves why the experimenter is asking them a question about Bilge/Gökçe's false belief using the verb for ‘think’ and not ‘falsely think’, given that the information necessary to make the inference is in common ground. Children might interpret the false belief question as pragmatically infelicitous, causing them to respond in a different manner than intended. For instance, children might interpret this question as asking only what they remembered about the contents of the container, which is not the correct response (see Rakoczy & Oktay‐Gür, 2020, for similar arguments regarding attribution of true belief in languages without a separate lexical item for falsely thinking).

We can think of two ways to test this hypothesis. First, consider what might happen if a second experimenter asks the test question, critically introducing this person after revealing the container is deceptive (so that the second experimenter does not have common ground with the child about the container's contents). If this hypothesis is correct, and children respond incorrectly because they find the situation infelicitous and thus confusing, then they should be more likely to respond correctly when a second experimenter asks the question. Second, consider what might happen if children were asked both false and true belief questions, pairing each with the more pragmatically appropriate verb. That is, if Turkish‐speaking children are first given an expected contents task (i.e. a true belief task), and tested on their knowledge of another's true belief using the verb ‘think’, it is possible that their performance on a measure of another's false belief would improve, particularly when the verb for ‘falsely think’ was used. This priming might be particularly salient if children are given explicit feedback about the correct answer using the appropriate lexical item (i.e. yes, the character <thinks> there are the expected contents in the box, after children respond correctly on a true belief trial…And what does the character <falsely think> is in the box with unexpected contents on a false belief trial?). Such priming might provide children with pragmatic support to respond appropriately (see Huang et al., 2018; Skordos et al., 2020, for related findings).

All three of these hypotheses make an auxiliary prediction, which is that performance on non‐linguistic measures of toddlers' false belief understanding (e.g. Onishi & Baillargeon, 2005) would be similar across cultures. We are unaware of any prior studies with Turkish‐speaking children that have used such non‐linguistic measures of false beliefs. Barrett et al. (2013), however, tested a non‐linguistic false belief measure with children from three traditional, non‐Western societies. One group was Salar children, whose language includes a lexical item for falsely think, similar to Turkish. These children and the children from the other two cultures, demonstrated performance comparable to that of children from Western societies, providing preliminary evidence supporting this hypothesis.

Finally, we have alluded to the possibility that the ‘collectivist’ pattern of development of theory of mind relates to the difference in performance between the self‐ and other question. An interesting point of consideration is to examine whether the distinction between the self‐ and other questions extends to cultures that exhibit the same ‘collectivist’ pattern. To our knowledge, differences in performance on the self‐ and other questions have not been systematically investigated in these cultures. It is possible that other cultures that exhibit the collectivist pattern of theory of mind development also show a difference in performance on the self‐ and other questions, particularly if those cultures endorse practices that emphasize ‘shared knowledge and mutual understanding of facts as documented in prior research on Turkish parental beliefs’ (Selcuk et al., 2018, p. 9). It is also possible that these findings are unique to Turkish culture because of the development of cultural practices or the evidential system unique to the language. We are currently exploring this possibility with both archival and novel data collection.

We also wish to highlight some limitations of the present study, which should be the subject for future investigations. First, the samples of children investigated here consisted mostly of 4‐ and 5‐year‐olds (particularly in Study 2), and relatively few 3‐year‐olds. This demographic distinction was due to the low enrolment rate for this age group in Turkish preschools (Ministry of National Education, 2024). Future studies should increase the representation of 3‐year‐olds in the sample. Second, language skills and inhibitory control measures are also associated with false belief performance (e.g. Carlson & Moses, 2001), but these variables were not considered. In particular, if Turkish children's interpretations of representational change and false belief questions differ based on pragmatic factors, then one might expect different correlational patterns between correct responses on these questions and measures of inhibition, but not of language. We are currently investigating this question in our laboratory.

To conclude, the present study showed that using a standard unexpected contents task, Turkish children showed superior performance on a representational change question about their own knowledge over a question about their understanding of others' false belief. Consistent with other investigations (e.g. Harris, 2012; Harris et al., 2017; Phillips et al., 2021; Sobel, 2023), we suggest that children's understanding of the representational nature of their own and others' belief states might have different developmental trajectories. Our data suggest that responses to these questions have different developmental trajectories in Turkish samples. It is possible that such differences emerge more generally across communities based on the diversity of linguistic and cognitive factors that relate to theory of mind development. We hope that other researchers interested in theory of mind development investigate potential differences between representational change and false belief understanding across cultures, not only to examine the generality of the present findings, but also to consider mechanisms for the diversity of theory of mind development.

## AUTHOR CONTRIBUTIONS

**Mesut Saçkes:** Conceptualization; investigation; writing – original draft; methodology; writing – review and editing; formal analysis; data curation. **Sonnur Işıtan:** Investigation; data curation; writing – original draft. **Kathy Cabe Trundle:** Writing – original draft; writing – review and editing. **David M. Sobel:** Writing – original draft; writing – review and editing; formal analysis; conceptualization; methodology; data curation.

## CONFLICT OF INTEREST STATEMENT

The authors declare no conflict of interest.

## Data Availability

The data and codes necessary to reproduce the analyses presented here and the materials necessary to attempt to replicate the findings are available from the corresponding author upon request. Analyses were not pre‐registered.

## Footnotes

^1^
Approximately 98% of the children passed the control question. This scoring system parallels that of Wellman and Liu (2004).
